# Dynamics of antigenemia and transmission intensity of *Wuchereria bancrofti* following cessation of mass drug administration in a formerly highly endemic region of Mali

**DOI:** 10.1186/s13071-016-1911-9

**Published:** 2016-12-03

**Authors:** Yaya I. Coulibaly, Siaka Y. Coulibaly, Housseini Dolo, Siaka Konate, Abdallah A. Diallo, Salif S. Doumbia, Lamine Soumaoro, Michel E. Coulibaly, Ilo Dicko, Moussa B. Sangare, Benoit Dembele, Modibo Sangare, Massitan Dembele, Yeya T. Touré, Louise Kelly-Hope, Katja Polman, Dominique Kyelem, Sekou F. Traore, Moses Bockarie, Amy D. Klion, Thomas B. Nutman

**Affiliations:** 1 Filariasis Unit, International Center of Excellence in Research, Faculty of Medicine and Odontostomatology, Point G, Bamako, Mali; 2 National Lymphatic Filariasis Elimination Program, Bamako, Mali; 3 Faculty of Medicine and Odontostomatology of Bamako, Bamako, Mali; 4 Filarial Program Support Unit, Liverpool School of Tropical Medicine, Liverpool, UK; 5 Department of Biomedical Sciences, Institute of Tropical Medicine, Antwerp, Belgium; 6 Neglected Tropical Diseases Support Center, Task Force for Global Health, Decatur, GA USA; 7 Department of Vector Biology, Liverpool School of Tropical Medicine, Liverpool, L3 5QA UK; 8 Laboratory of Parasitic Diseases, National Institute of Allergy and Infectious Diseases, National Institutes of Health, Bethesda, MD USA

**Keywords:** *Wuchereria bancrofti*, Transmission assessment survey, *Anopheles gambiae* complex, Mass drug administration, Post-MDA surveillance

## Abstract

**Background:**

After seven annual rounds of mass drug administration (MDA) in six Malian villages highly endemic for *Wuchereria bancrofti* (overall prevalence rate of 42.7%), treatment was discontinued in 2008. Surveillance was performed over the ensuing 5 years to detect recrudescence.

**Methods:**

Circulating filarial antigen (CFA) was measured using immunochromatographic card tests (ICT) and Og4C3 ELISA in 6–7 year-olds. Antibody to the *W. bancrofti* infective larval stage (L3) antigen, Wb123, was tested in the same population in 2012. Microfilaraemia was assessed in ICT-positive subjects. *Anopheles gambiae* complex specimens were collected monthly using human landing catch (HLC) and pyrethrum spray catch (PSC). *Anopheles gambiae* complex infection with *W. bancrofti* was determined by dissection and reverse transcriptase polymerase chain reaction (RT-PCR) of mosquito pools.

**Results:**

Annual CFA prevalence rates using ICT in children increased over time from 0% (0/289) in 2009 to 2.7% (8/301) in 2011, 3.9% (11/285) in 2012 and 4.5% (14/309) in 2013 (trend *χ* ^2^ *=* 11.85, *df* =3*, P* = 0.0006). Wb123 antibody positivity rates in 2013 were similar to the CFA prevalence by ELISA (5/285). Although two *W. bancrofti*-infected *Anopheles* were observed by dissection among 12,951 mosquitoes collected by HLC, none had L3 larvae when tested by L3-specific RT-PCR. No positive pools were detected among the mosquitoes collected by pyrethrum spray catch. Whereas ICT in 6–7 year-olds was the major surveillance tool, ICT positivity was also assessed in older children and adults (8–65 years old). CFA prevalence decreased in this group from 4.9% (39/800) to 3.5% (28/795) and 2.8% (50/1,812) in 2009, 2011 and 2012, respectively (trend *χ* ^2^ *=* 7.361, *df* =2*, P* = 0.0067). Some ICT-positive individuals were microfilaraemic in 2009 [2.6% (1/39)] and 2011 [8.3% (3/36)], but none were positive in 2012 or 2013.

**Conclusion:**

Although ICT rates in children increased over the 5-year surveillance period, the decrease in ICT prevalence in the older group suggests a reduction in transmission intensity. This was consistent with the failure to detect infective mosquitoes or microfilaraemia. The threshold of ICT positivity in children may need to be re-assessed and other adjunct surveillance tools considered.

## Background

Lymphatic filariasis (LF) is a public health problem in 71 countries and is associated with marked morbidity and disability [1]. To eliminate LF by 2020, the Global Program to Eliminate LF (GPELF) adopted strategies based on two pillars: annual mass drug administration (MDA) to all eligible residents of the endemic communities and morbidity management [2]. MDA is aimed at interrupting LF transmission through clearing of peripheral blood microfilariae that prevent human-to-human vector-borne transmission [2].

As Bancroftian filariasis was found to be endemic in all eight administrative districts of Mali, ranging from 1% in Timbuktu (northern part of Mali) to > 18% in Sikasso (southern part of the country) [3], annual MDA using ivermectin and albendazole was initiated sequentially starting from the most highly endemic district in the country [3]. Sentinel sites were established in Sikasso as part of a multi-country initiative to assess LF transmission during and after stopping MDA. The baseline data and the impact of six rounds of MDA on human infection and potential transmission in this sentinel site have been previously reported [4].

The current study reports data collected to assess transmission after MDA was stopped in 2007 (after seven rounds of MDA). Although this study was initiated prior to the formal WHO recommendations for transmission assessment surveys (TAS), which require demonstration of an infection rate of < 1% in > 400 children aged 6–7 years using the immunochromatographic card test (ICT) to document interruption of transmission [5], a similar approach was taken using ICT testing of children aged 6–7 years. ICT testing of a cohort of children ≥ 8 years old and adults and entomological assessment of LF transmission were performed. Finally, the use of several additional methods (Og4C3 ELISA; Polymerase Chain Reaction (PCR) targeting *Wuchereria bancrofti* DNA; and *W. bancrofti* infective larval stage specific antigen Wb123-based IgG4 immunoassays) to assess transmission interruption in this previously highly LF-endemic region (Sikasso) of Mali was explored. Our data support an integrated approach to surveillance.

## Methods

### Study sites

The study area comprised six villages in Sikasso district: Gondaga, Dozanso, Missasso, N’torla, Niatanso and Zanadougou. These villages are located in the rural commune of Kolokoba that is located 332 km southeast of Bamako the capital city. *Wuchereria bancrofti* infection prevalence as assessed by the detection of CFA using ICT prior to MDA was 46% [4]. This area is also endemic for *Mansonella perstans*, but not *Onchocerca volvulus* infection. Based on 2012 census data, the total population of the 6 villages was 5044. The study villages had undergone 7 annual rounds of MDA prior to its cessation in 2008, at which time the CFA prevalence had decreased to 0/760 children tested and the *Anopheles gambiae* complex mosquitoes showed an infection rate of 0.04% and an infectivity rate of 0.02% that were felt to be incompatible with active LF transmission [6]. There was a mean programmatic coverage rate based on the total population of 75.6% that varied from 67 to 78% [6]. A year after cessation of MDA (in 2009), no infected 6–7 year-old children were found among the 120 tested in the six villages.

### Study design

**Fig. 1:**
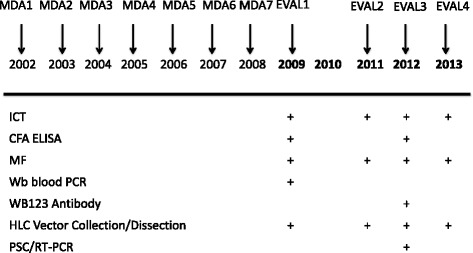
Time line of treatment, EVAL, and monitoring. The years in which MDA and EVAL surveys were performed are shown by the *arrows* and times at which monitoring tools in support of EVAL are shown by the + sign

As post-MDA surveillance, a yearly cross-sectional parasitological assessment of all children 6–7 years of age and all eligible older volunteers aged 8 years and above was performed in July from 2009 to 2012. In addition, a monthly entomological assessment of LF transmission (from July to December) was conducted in the six study villages in 2009, 2011 and 2013. In 2013, only children aged 6–7 years were tested with the ICT, along with a thick smear from night blood. Infective stage *W. bancrofti* larvae (L3) were assessed in mosquitoes using an L3-specific reverse transcriptase PCR (RT-PCR) technique as previously described [7]. The study design is illustrated in Fig. 1. EVAL refers to the ensemble of surveillance testing performed in any given year.

### Parasitological and serological assessments

Infection status was assessed using the ICT card test for the detection of circulating *W. bancrofti* antigen (Allere, Portland, ME, USA). Dried blood spots were collected for additional laboratory analyses. Microfilaraemia was assessed by finger prick at night (between 22:00 and 02:00 h) among ICT-positive volunteers using a calibrated thick smear. Yearly parasitological studies were conducted in July, at the beginning of the transmission period, except in 2009 when, for logistical reasons, this assessment was performed in October. Because of the concern of potential transmission, additional diagnostic tests were performed on eluted blood spots from the 6–7 year-old children in 2012, namely the Og4C3 ELISA (Tropbio Townsville, Australia) and ELISA testing for antibodies to Wb123 as previously described [8].

### Entomological assessment

Each month, a 12-day entomological survey was conducted in the six villages to assess the village wide *W. bancrofti* transmission patterns during the LF transmission period in Mali from July to December [4]. Two human landing catch (HLC) sessions were organized per month and per village. Two collectors worked inside each of four collection rooms per session.

Because *An. gambiae* is endophilic, collections were performed indoors to maximize yield. A total of 72 collection rounds were undertaken with the HLC. The collection was done from 18:00 to 06:00 h, and for ethical reasons, the collectors were replaced at midnight at each collection site. All *An. gambiae* and *An. funestus* complexes collected were freshly dissected for parity status based on techniques previously described [9, 10] and for infection (any larval stage) and infectivity (L3 stage) status by individual mosquito dissection as previously described [11].

In 2012, the PSC (Pyrethrum Spray Catch) was used to collect mosquito vectors in addition to the HLC using Premium®, a pyrethrinoid-based insecticide, in 30 randomly selected rooms per village in each of the six collection months. During each of the 36 PSC collection rounds, the number of persons sleeping in each visited room was recorded on the mosquito collection sheet. The collected mosquitoes during the PSC were pooled (1 to 20 mosquitoes) in the field and stored in tubes containing RNA later and sent to Smith College for *W. bancrofti* RNA detection by RT-PCR as previously described [7].

For PSC, the monthly biting rate was determined by dividing the number of fed and half-gravid female *Anopheles* collected in a room by the number of sleepers in the room the night before the collection multiplied by 30 [12]. The annual biting rate (ABR) was the sum of all the monthly biting rates calculated over the year [12]. From HLC-collected *Anopheles*, the parameters were determined as previously reported [12, 13].

### Sampling

The present study predated the official WHO guidelines for TAS [5]. Because the evaluation unit was small (<300 children aged 6–7 years), all of the available eligible children were screened.

### Data analysis

The collected data were entered using Microsoft Access 2007 and analysed using Graph Pad prism version 5 and Statistical Package for Social Sciences (SPSS) version 20. To compare the infection prevalences between villages or mosquito species, we used the Pearson *χ* ^2^ or the Fisher’s exact test, if necessary. The trend *χ* ^2^ was used to test the statistical significance of any frequency or proportion’s trend over time.

## Results

### Study demographics

We assessed 289 children aged 6–7 years in 2009, 301 in 2011, 285 in 2012 and 309 in 2013. Concomitantly, available older children and adults were assessed in 2009 (*n* = 800), 2011 (*n* = 795), and 2012 (*n* = 1812) (Tables 1 and 2). In 2013, testing of older children and adults was restricted to those who were positive by ICT in 2012 (*n* = 50). Although the sizes of the six study villages differed, the study populations within the villages were quite well balanced in terms of gender within both the 6 to 7 year-old children and the ≥ 8 year-olds throughout the study period (Table 2).

**Table 1:** Sampling according to different activities per year

	Study human sample			Mosquito collection		
Year	Total population	6–7 years old	≥ 8 years	Number collected	Technique used	Number of collection rounds
2009	4,431	289	800	4,448	HLC	72
2011^a^	4,761	301	795	2,962	HLC	72
2012	5,044	285	1,812	7,168/1,907	HLC/PSC	72/36^b^
2013^c^	5,225	309	50	nd	nd	nd

*Abbreviation*: *nd* not done

^a^In 2011, a random sample of 92 subjects from the 6 villages was tested with Wb123 ELISA

^b^In 2012, the six villages were visited once a month from July to December (collection in 30 rooms per visit per village)

^c^In 2013 the 50 subjects ≥ 8 years tested were the ones found positive using ICT in 2012

**Table 2:** Characteristics of the study population per village throughout the surveillance period in the 6 study villages of the Sikasso district

Village	6–7 years		8 years and above			Overall
	M/F	Total	M/F	Median age (Range)	Total	
Survey 1 in 2009						
Dozanso	20/29	49	60/73	34 (12–79)	133	182
Missasso	26/20	46	64/94	40 (15–76)	158	204
Gondaga	22/21	43	55/64	33 (12–75)	119	162
Niatanso	30/24	54	91/106	31 (12–69)	197	251
N’Torla	23/16	39	50/49	37 (12–72)	99	138
Zanadougou	28/30	58	31/63	37.5 (13–77)	94	152
Total	149/140	289	351/449	35 (12–79)	800	1,089
Survey 2 in 2011						
Dozanso	21/17	38	42/71	32 (15–82)	113	151
Missasso	22/31	53	51/99	35 (15–86)	150	203
Gondaga	21/17	38	58/73	29 (15–84)	131	169
Niatanso	25/29	54	73/60	31 (15–82)	133	187
N’Torla	35/26	61	53/80	31 (15–88)	133	194
Zanadougou	26/31	57	49/86	31 (15–89)	135	192
Total	150/151	301	326/469	38 (15–89)	795	1,096
Survey 3 in 2012						
Dozanso	20/16	36	95/137	32 (15–82)	232	268
Missasso	21/27	48	101/171	33 (15–79)	272	320
Gondaga	27/21	48	100/177	28 (15–85)	277	325
Niatanso	26/25	51	134/182	28 (15–83)	316	367
N’Torla	22/15	37	127/208	30 (15–89)	335	372
Zanadougou	34/31	65	137/243	30 (15–80)	380	445
Total	150/135	285	694/1,118	30 (15–89)	1,812	2,097
Survey 4 in 2013						
Dozanso	24/24	48	8/15	41 (8–75)	23	73
Missasso	26/21	47	1/4	38 (31–68)	5	52
Gondaga	30/25	55	0/5	28 (8–58)	5	60
Niatanso	32/23	55	1/3	25.5 (8–63)	4	60
N’Torla	18/24	42	1/6	46 (24–66)	7	49
Zanadougou	31/31	62	4/2	29 (8–58)	6	68
Total	161/148	309	15/35	38 (8–75)	50	359

*Abbreviation*: *M/F* male/female

### CFA and Wb123 antibody prevalence assessment over the surveillance period

The CFA prevalence in 6–7 year-old children increased significantly over the surveillance period, from 0% (0/289) in 2009 to 2.7% (8/301) in 2011 and 4.5% (14/309) in 2013 (trend *χ* ^2^ = 12.80, *df* = 3, *P* = 0.0003) (Table 3). In contrast, there was a significant decrease in CFA positivity over the study period in the ≥ 8 year-olds, from 4.9% (39/800) in 2009 to 3.5% (28/795) in 2011, to 2.8% (50/1,812) in 2012 (trend *χ* ^2^ = 697.8, *df* = 2, *P* = 0.0001). Whereas none of the ICT-positive 6–7 year-olds had detectable microfilaraemia, 1 of 39 (2.6%) individuals in the older group was microfilaraemic in 2009, and 3/36 (8.3%) were microfilaraemic in 2011. In 2012, none of the 50 ICT-positive older subjects was microfilaraemic (Table 3). Forty-four of the previously ICT-positive older subjects, as well as 6 of the 6–7 year-olds who were ICT-positive and 8 years old at the time of the 2013 survey, were reassessed in 2013. None of the 28 subjects who remained ICT-positive in 2013 had detectable microfilaraemia (data not shown). Positivity rates for both the Og4C3 ELISA for CFA and testing for antibodies to the *W. bancrofti*-specific antigen, Wb 123, were similar to the results obtained using the ICT tests (*χ* ^2^ = 3.52, *df* = 2, *P* = 0.173).

**Table 3:** Circulating filarial antigen (CFA) and microfilaraemia prevalence rates within 6–7 year-old children and those of 8 years and above from 2009 to 2013

		Survey 1 (2009)	Survey 2 (2011)	Survey 3 (2012)^a^	Survey 4 (2013)
Sample size and target	Targeted sample size	1,107	1,107	2,530	372
	Total population	4,431	4,761	5,044	5,225
	Number tested (*n*)	1,089	1,096	2,097	359
ICT	≥ 8 years % Positive (*n*/*N*)	4.9% (39/800)	3.5% (28/795)	2.8% (50/1,812)	
	[95% CI]	[3.53–6.67]	[2.40–5.12]	[2.08–3.65]	–
	6–7 years % Positive (*n*/*N*)	0% (0/289)	2.7% (8/301)	3.9% (11/285)	4.5% (14/309)
	[95% CI]	[0.00–1.64]	[1.24–5.37]	[2.04–7.00]	[2.60–7.66]
Mf	≥ 8 years % Positive (*n*/*N*)^b^	2.6% (1/39)	10.7% (3/28)	0% (0/50)	
	[95% CI]	[0.06–13.48]	[2.81–29.37]	[0.00–8.89]	–
	6–7 years % Positive (*n*/*N*)^b^	0	0% (0/8)	0% (0/11)	0% (0/14)
	[95% CI]		[0.00–40.23]	[0.00–32.15]	[0.00–26.76]
PCR	≥ 8 years % Positive (*n*/*N*)	5.13% (2/39)	np	np	np
	[95% CI]	[0.89–18.63]			
	6–7 years % Positive (*n*/*N*)	0	np	np	np
	[95% CI]				
Wb123	≥ 8 years % Positive (*n*/*N*)	np	np	4.7% (2/43)	nd
	[95% CI]			[0.81–17.06]	
	6–7 years % Positive (*n*/*N*)	np	np	1.8% (5/285)	nd
	[95% CI]			[0.65–4.27]	
Og4C3	≥ 8 years ICT % Positive (*n*/*N*)	np	np	4% (2/50)	np
	[95% CI]			[0.70–14.86]	
	6–7 years % Positive (*n*/*N*)	np	np	1.8% (5/285)	np
	[95% CI]			[0.65–4.27]	

*Abbreviations*: *ELISA* enzyme-linked immuno-sorbent assay, *ICT* immunochromatographic card test, *ICT+* ICT positive, *Mf* microfilaraemia, *n* number positive, *N* number examined, *nd* not done, *np* not planned, *PCR* polymerase chain reaction, *Wb123* filarial antibody test

^a^In 2012, the ELISA test was done on all the children and the 50 ICT positive adults

^b^Only the ICT positive subjects were tested for Mf

### Entomological assessment

The number of mosquitoes collected using the HLC over the study period is detailed in Table 4. The highest ABR using the HLC was 374 bites per person in 2012 and the lowest was in 2011 with 155 bites per person. Among the dissected mosquitoes, the parity rates were significantly different between the 3 yearly entomological surveys with 84% (3,675/4,380) in 2009, 84% (2,406/2,853) in 2011 and 88% (5,032/5,718) in 2012 (*χ* ^2^ = 40.76, *df* = 2, *P* <10^-4^). In 2009, two (0.05%) filaria-infected *Anopheles* females were detected (Table 4) without any infective larval stage recovered. In 2011 and 2012, no *W. bancrofti* larvae were found in the dissected mosquitoes (data not shown).

**Table 4:** Annual variation of mosquito densities and biting rates over the surveillance period from 2009 to 2012

Collection method	Years	Species	No. of mosquitoes collected	No. of mosquitoes dissected Frequency [95% CI]	ABR	Parity Frequency [95% CI]	Infection Frequency [95% CI]
HLC	2009	GA	4,443	4,375	232	3,671	2
				98.47 [98.05–98.8]		83.9 [82.78–84.98]	0.05 [0.01–0.18]
		FU	5	5	0	4	0
				100 [46.29–100]		80 [29.88–98.94]	
		PH	0	0	0	0	0
		RU	0	0	0	0	0
	2011	GA	2,911	2,803	152	2,364	0
				96.29 [95.52–96.93]		84.34 [82.92–85.65]	
		FU	3	3	0	3	0
				100 [31.00–100]		100 [31.00–100]	
		PH	39	38	2	30	0
				97.44 [84.92–99.87]		78.95 [62.22–89.86]	
		RU	9	9	1	9	0
				100 [62.88–100]		100 [62.88–100]	
	2012	GA	7,138	5,691	368	5,006	0
				79.82 [78.86–80.74]		88.9 [88.05–89.70]	
		FU	3	3	0	3	0
				100 [31.00–100]		100 [31.00–100]	
		PH	23	23	1	22	0
				100 [77.08–100]		94.1 [69.23–99.69]	
		RU	1	1	0	1	0
				100 [5.46–100]		100 [5.46–100]	
PSC	2009		nd	nd	nd	nd	nd
	2011		nd	nd	nd	nd	nd
	2012	*An*. spp.	1,907	115^a^	12^b^	nd	0

*Abbreviations*: *An*. spp. *Anopheles* species, *HBR* human biting rate, *HLC* human landing catch, *FU Anopheles funestus, GA Anopheles gambiae, PH Anopheles pharaoensis, PSC* Pyrethrum spray catch, *RU Anopheles rufipes, nd* not done

^a^Number of pools of 20 mosquitoes tested with the RT-PCR

^b^The number of half gravid and blood fed mosquitoes divided by the number of sleepers in the rooms visited the night before the collection

With the PSC method during the 6 months of collection in 2012, 1907 mosquitoes were collected and the ABR was 100 bites per person per year. The number of mosquitoes collected with the PSC technique was 3.75 times less than that collected with the HLC in 2012. Moreover, both the infection and infectivity of the PSC-collected mosquitoes were 0 (Table 4). Of note, *An. gambiae* complex was the most frequent vector comprising more than 99% of the active vector fauna each year as compared to *An. funestus* complex (data not shown)*.*

We observed the highest vector density (12 mosquitoes per person per night) in 2012 with 7165 mosquitoes collected by 576 collectors. This density was 2.4 times higher than that in 2011 (2962 mosquitoes) and 1.6 times more than that in 2009 (4448 mosquitoes). Of the 2962 and 7165 mosquitoes collected in 2011 and 2012, respectively, the frequencies of *An. pharaoensis* varied from 1.31% in 2011 to 0.32% in 2012 while the frequencies of *An. rufipes* varied from 0.30% in 2011 to 0.01% in 2012. These species were very rare during the previous collection years in this area and were never found to be infected with *W. bancrofti* (Table 4).

## Discussion

The current study investigated the LF transmission patterns following cessation of MDA during the surveillance period from 2009 to 2013 in six neighbouring previously highly LF endemic villages in the Sikasso region in Mali. In 2008, after seven rounds of MDA, the *W. bancrofti* microfilaraemia and ICT positivity in children (6–7 years) was reduced to 0%. By 2011 and 2012, the prevalence of ICT-positivity in 6–7 year-old children showed an increase, although microfilaraemia was not detected. Despite a steady increase in CFA prevalence in 6–7 year-old children, there was a marked decrease in CFA prevalence rates over the same 5 year period among those ≥ 8 years of age (trend *χ* ^2^ = 7.361, *df* = 3, *P* = 0.0067). This decrease is consistent with attrition over time of established worms. Despite the increasing CFA prevalence in children, our data are most consistent with interruption of LF transmission based on the absence of detectable microfilaraemia, the lack of infective *Anopheles*, and the decreased CFA prevalence in the older age group. Nonetheless, close monitoring in areas of previously high transmission is necessary to detect early resurgence of transmission and to generate data that may guide and improve the LF elimination process.

When prevalence was estimated using different tools (Og4C3 ELISA and Wb123 immunoassays) at a single time point (2012), ICT consistently gave a higher prevalence rate compared to the two other tests, although the differences in prevalence were not statistically significant. Higher prevalences using ICT compared to Og4C3 ELISA was also observed in Togo during a school-based TAS conducted 3 years after stopping MDA [14], although the reasons for this are unclear. *Loa loa* microfilaraemia has been shown to be associated with ICT-positivity at both the community and individual levels [15, 16]; however, the same studies showed no association between ICT-positivity and the prevalence of *M. perstans*, the only other filarial parasite endemic in the study area [17].

Re-emergence of infection after just a few years of surveillance has been reported in Nigeria in some but not in all districts [18]. In India after 10 years following MDA implementation, new infection among children was also reported [19]. Using 6–7 year-old children as the sentinel population makes sense in the Malian context because this group remains in the villages, whereas many adults travel from place to place because of seasonal migration for agriculture and may acquire infection in areas that have not yet started MDA [20].

The approach to post-MDA surveillance is still being perfected. Antibody testing (e.g. Wb123) has been proposed as a potential better tool than antigen testing for the early identification of on-going transmission, as antibody positivity typically occurs months prior to positivity in adult antigen-based circulating antigen testing [21–23]. As there was good concordance between Wb123 prevalence and that of the CFA testing in the children (see Table 1) and with both tests now being point of care (POC) [8, 24], it is possible that the Wb123 rapid diagnostic test may be considered as a major surveillance tool in the near future.

Although screening of vector populations for the presence of infective larvae has been one of the 2 pillars of assessing transmission interruption in onchocerciasis [25, 26], its widespread use in LF has not taken hold to date. However, using both standard (dissection) and molecular techniques on both HLC and PSC collected mosquitoes (*n* = 9072) only a few positives were found (and only just after the cessation of MDA). This is probably due to the drastic reduction of microfilaraemia prevalence after the seven consecutive MDA treatments and to the relatively low number of mosquitoes collected and the low sensitivity of the dissection [7]. Since RT-PCR, a more sensitive method to detect infective stage L3 larvae in the vector, is available [7], screening of larger numbers of mosquitoes and pool screen-based molecular techniques will need to be assessed.

The observation that *An. pharaoensis* and *An. rufipes* were more frequently biting humans and their identification as secondary vectors of *W. bancrofti* in West Africa [27], raises the possibility that transmission can be sustained by a number of vectors other than the most prevalent (*An. gambiae* complex). The rain pattern in 2012 (frequency and abundance) likely played a role in the increased vector density, as well as in the increase in *An. pharaoensis* and *An. rufipes* frequencies [28, 29]. However, what is needed is an adequately designed prospective study of *W. bancrofti* transmission dynamics and vector control in this region of Mali. In addition, HLC was much more effective at collecting *Anopheles* than PSC; because of potential ethical issues related to HLC [30], better collection methods are needed.

With very low human infection and vector infectivity rates, there is no evidence that *W. bancrofti* transmission has re-emerged in the study villages in the present study [5, 12]. Nevertheless, new entomological studies are needed to understand transmission dynamics in the context of post MDA surveillance. Mosquito vectors transmit *W. bancrofti* in two primary patterns, limitation and facilitation. Limitation is typically exhibited by *Culex* species and allows more efficient L3 development when microfilaraemia loads are low. Conversely, facilitation (usually exhibited by *Anopheles* species) leads to decreased numbers of developing L3 when microfilaraemia loads are low. Because limitation of *An. gambiae* (*sensu stricto*) has been observed in Ghana [31], it should also be assessed in other geographic locations (e.g. Mali) given the possibility of adaptation or specific mutation that can modify mosquito’s transmission pattern [32]. From our previous studies, in the same area, WHO criteria were met but the mosquitoes were still infective (infectivity rate of 0.02%) when the MDA was stopped [6]. Taking into account the entomological data and determining a threshold could be beneficial to be able to safely stop MDA in highly LF endemic areas.

Despite a dramatic and stable decrease in the prevalence of infection in the older age groups and in mosquitoes 5 years following the cessation of MDA in six villages previously highly endemic for LF, a significant increase in the prevalence of LF antigenemia as assessed by ICT occurred among 6–7 year-old children. Although the ICT prevalence in this age group met WHO criteria for restarting MDA (> 2% ICT-positive) [5], the prevalence using the Og4C3 ELISA and Wb123 antibody ELISA were below the threshold. Furthermore, the observed prevalence increase within this group contrasted with the entomological data that showed an absence of LF transmission and the absence of microfilaraemia in all individuals tested.

## Conclusions

Using a set of LF testing methods (ICT, Wb123, Og4C3 ELISA, and vector surveillance), we demonstrated differences among the various techniques considered important for post-MDA assessments. Our data suggest, nevertheless, that an integrated assessment strategy that combines serologic- and vector-based techniques may be useful in the assessment of transmission interruption following cessation of MDA in LF-endemic areas.

## Abbreviations

ABR: Annual biting rate
CFA: Circulating filarial antigen
DNA: Deoxyribonucleic acid
GPELF: Global program to eliminate lymphatic filariasis
HLC: Human landing catch
ICT: Immunochromatographic card tests
IgG4: Immunoglobulins g4
LF: Lymphatic filariasis
MDA: Mass drug administration
NIAID: National Institute of Allergy and Infectious Diseases
NIH: National Institute for Health
POC: Point of care
PSC: Pyrethrum spray catch
RNA: Ribonucleic acid
RT-PCR: Reverse transcriptase polymerase chain reaction
SPSS: Statistical package for social sciences
TAS: Transmission assessment surveys
UNDP: United Nation Development Programme
WHO: World Health Organization
